# Clinicians' Experiences in Responding to Mental Health Crisis Among People Experiencing Homelessness in Ireland

**DOI:** 10.1111/jpm.70116

**Published:** 2026-03-03

**Authors:** John P Gilmore, Shubhangi Karmakar, Krupa Joy, Adam Chukwu

**Affiliations:** ^1^ University College Dublin School of Nursing, Midwifery and Health Systems University College Dublin Dublin Ireland

**Keywords:** health inequalities, homelessness, inclusion health, mental health, nursing, trauma‐informed care

## Abstract

**Introduction:**

Homelessness is rising in Ireland, and people experiencing homelessness face disproportionately poor mental health outcomes due to intersecting structural and psychosocial factors. Access to mental healthcare remains limited, leaving generalist services to manage increasing need, particularly during crises.

**Aim:**

To explore healthcare professionals' experiences of managing mental health crisis episodes among people experiencing homelessness.

**Method:**

A descriptive qualitative study informed by critical realism. We used semi‐structured interviews with 14 healthcare professionals, including GPs, nurses, a psychotherapist, a hospital consultant, and service leaders. Reflexive Thematic Analysis was used to identify key patterns.

**Results:**

Five themes were identified: (1) barriers to accessing mental health services; (2) the role of trust and therapeutic relationships; (3) trauma‐informed and holistic approaches to care; (4) the need for stability in housing, healthcare, and substance use; and (5) systemic and policy failures.

**Discussion:**

Generalist professionals are increasingly required to provide complex mental health support in a fragmented system that often excludes the most vulnerable.

**Limitations:**

Findings reflect professional perspectives from a small sample and are not generalisable.

**Implications:**

Trauma‐informed, flexible, and integrated services are essential to support equitable care.

**Recommendations:**

Develop inclusive, housing‐linked mental health services that embrace harm reduction and reject rigid eligibility criteria.

## Summary

**What is known on the subject?:**

○ People experiencing homelessness have worse mental health and often face barriers to accessing care.
○ Services are often fragmented and difficult to navigate, particularly for those with complex needs.
○ Often the specialist support required for those experiencing crisis in their mental health is left up to general healthcare services for people experiencing homelessness.

**Originality:**

○ This study shares the views and experiences of healthcare professionals in general services that support people experiencing homelessness with their mental health.
○ It highlights the limitations of current systems and offers insight into more flexible, compassionate approaches.
○ Participants advocate for trauma‐informed and harm‐reduction‐based models that recognise the realities of homelessness.

**Significance:**

○ Rigid service rules can prevent access to care for people who need it most.
○ Highlights the need for trust, consistency, and respect in mental health services.
○ Findings can inform more inclusive and effective services for people experiencing homelessness.

**Rigour:**

○ Each interview was recorded and written out word‐for‐word to make sure we captured exactly what people said.
○ We used Reflexive Thematic Analysis to carefully identify patterns and themes in what participants said.
○ We continued interviewing people until no new ideas were coming up, showing the findings were well developed and based on rich data.

**Relevance Statement:**

This study is highly relevant to mental health nursing practice, as it explores the real‐world challenges of providing mental health care to people experiencing homelessness, an often overlooked group with complex, overlapping needs. It highlights the importance of trauma‐informed, relational, and harm‐reduction approaches, all central to contemporary mental health nursing values. Findings offer clear implications for improving therapeutic engagement, service accessibility, and policy reform.

## 1 Introduction

Across high‐income countries, homelessness is increasingly recognised as a major public health concern, shaped by structural factors including housing market pressures, welfare retrenchment, service fragmentation, and the criminalisation of poverty. In Europe, homelessness continues to rise, with migrants, women, children, and ethnic minorities particularly affected (European Commission 2022); Ireland is notably experiencing one of the most severe national spikes, with the number of individuals in emergency accommodation more than doubling since 2015 and family homelessness increasing by over 300% (Allen et al. 2020; Department of Housing, Local Government and Heritage 2022; Long et al. 2019).

Homeless populations experience significantly poorer physical and mental health, driven by intersecting structural, environmental, and psychosocial factors such as poverty, trauma, stigma, and limited access to care (Carter‐Pokras and Baquet 2002; Fazel et al. 2014; Ingram et al. 2023). In Ireland, the absence of routine data linkage between homelessness and health further obscures this population in service planning (McNeill et al. 2022; Ivers et al. 2019). Rates of psychological distress, severe mental illness, trauma, and substance use are markedly higher among people experiencing homelessness (Gutwinski et al. 2021; Ingram et al. 2023). Despite national policy commitments to community‐based mental health services, Ireland lacks dedicated supports for this group, and those without a fixed address are often excluded from care pathways (McNeill et al. 2022). As a result, many must rely on overstretched general and primary care services for mental health support.

Specialist primary care and general health services for people experiencing homelessness are designed to address broader health inequalities and provide low‐threshold, person‐centred care in accessible settings. These services operate within a wider health system that often struggles to respond effectively to the realities of poverty, social exclusion, and complex co‐morbidity (Siersbaek et al. 2023). As the prevalence of untreated or poorly managed mental health issues rises among people who are homeless, clinicians working in these frontline settings are increasingly required to manage acute mental health episodes as part of routine care (Moloney et al. 2022).

Medical care in Ireland is delivered through a mixed public–private healthcare system, with publicly funded services provided by the Health Service Executive. Access to free primary and hospital medical care is largely mediated through the income‐based Medical Card scheme, with those not eligible often facing out‐of‐pocket costs for GP services.

In Ireland, access to secondary mental health services often depends on having a fixed address, access to a GP, and the ability to engage with standard referral pathways, all of which may be compromised by the experience of homelessness. Emergency departments may provide short‐term responses but lack continuity, while community mental health teams are frequently under pressure and may not be equipped to respond flexibly to people with unstable housing, ongoing substance use, or complex psychosocial needs (Mcloughlin et al. 2021). As a result, general health and primary care providers become de facto mental health crisis responders.

Clinicians and service leaders working in these contexts often describe a deep commitment to their patients, grounded in trust, persistence, and a recognition of the structural barriers people face. However, they also highlight the emotional and ethical strain of consistently working beyond their remit, navigating risk without adequate psychiatric support, and bearing the weight of unsupported decision‐making (Davies and Wood 2018).

This study aimed to explore healthcare professionals' experiences of providing mental health support to people experiencing homelessness, focusing on how acute deterioration is managed, the challenges involved, and the systemic factors shaping care. While grounded in the Irish health system, the findings may offer insights relevant to other high‐income countries facing similar structural pressures and service gaps.

## 2 Methodology

A qualitative descriptive design (Doyle et al. 2020) was used to provide a broad account of the phenomenon. We adopted an ontological position of Critical Realism, interpreting participant responses as mediated reflections of reality, shaped by and embedded within their cultural and professional contexts. A critical realist ontology recognises the complexity of social phenomena, such as the experience of supporting mental health during homelessness, by acknowledging the existence of underlying structures and mechanisms that shape observable events (Haigh et al. 2019; Bhaskar 1975). It provides a framework for understanding how these deeper layers of reality interact with human knowledge and experience.

We used semi‐structured interviews to explore the experiences of healthcare professionals providing mental health support to people experiencing homelessness, particularly in relation to acute or crisis mental health deterioration. Fourteen participants were recruited using purposive sampling, based on their professional roles and experience with this population. The research team comprised a nurse researcher with significant experience working with marginalised communities, two mental health nurses, and a psychiatry trainee, supported by an external advisory group. The interview scheduled is available as supplementary mater (Data S1).

Participants included 12 clinicians, four General Practitioners (GPs), three Specialist Nurses, three Nurse Managers, one Hospital Consultant, and one Psychotherapist. Two service leaders were also interviewed to provide systems and service‐level insights, enabling a broader understanding of the challenges and practices across clinical and organisational domains. Participants worked across various settings including general primary care services, primary care services specifically for marginalised communities, and tertiary hospital settings. Full participant details are available below (Table 1).

**TABLE 1:** Participant characteristics.

Participant No.	Role	Gender	Location	Years in homeless health
1	GP	Male	Dublin	5–10 years
2	GP	Male	Mid West	5–10 years
3	GP	Female	Dublin	> 20 years
4	GP	Female	Dublin	5–10 years
5	Consultant	Female	Dublin	5–10 years
6	Nurse Manager	Female	South of Ireland	5–10 years
7	Nurse Manager	Female	Dublin	5–10 years
8	Nurse Manager	Female	Dublin	< 5 years
9	Specialist Nurse	Female	South of Ireland	5–10 years
10	Specialist Nurse	Female	Dublin	10–20 years
11	Specialist Nurse	Female	Dublin	5–10 years
12	Psychotherapist	Female	Dublin	5–10 years
13	Service Leader	Female	Dublin	> 20 years
14	Service Leader	Female	Dublin	10–20 years

Data collection occurred between January and April 2024. Interviews were conducted by two of the researchers (JG, AC), either in person (*n* = 4) or online via Zoom (*n* = 10), depending on participant preference and availability. Each interview lasted 30–60 min and followed a semi‐structured guide designed to elicit reflective accounts of participants' experiences in managing mental health crises among people experiencing homelessness. All interviews were audio‐recorded with consent and transcribed verbatim, ensuring accuracy and depth of data.

Data analysis was conducted manually by the lead researcher, using Reflexive Thematic Analysis (RTA), following the six‐phase approach outlined by Braun and Clarke (2006, 2012). This approach enabled a flexible yet rigorous process of familiarisation, coding, theme development, and refinement. The reflexive orientation acknowledges the researcher's active role in interpretation and supports the generation of rich, nuanced themes grounded in the data. Thematic saturation was reached, with no new codes or concepts emerging during the final stages of analysis. Five key themes were developed that captured recurring patterns and meaningful insights across participant accounts.

Ethical approval was granted by University College Dublin Human Research Ethics Committee (Reference: LS‐LR‐23‐25‐Gilmore), and all participants provided written informed consent prior to participation.

## 3 Findings

The interviews revealed profound systemic and structural barriers that prevent people experiencing homelessness from accessing timely, appropriate, and compassionate healthcare, particularly mental health services, highlighting a fragmented system that often excludes the very individuals who need it most. The coding tree is exemplified below (Table 2).

**TABLE 2:** Coding tree.

Theme	Subthemes
Barriers to accessing healthcare and mental health services	Eligibility criteria and catchment‐based exclusions Dual diagnosis and abstinence‐based thresholds Stigma and discrimination in healthcare Disjointed service coordination
Building trust and therapeutic relationships	Importance of consistency and persistence Flexibility and informal approaches Challenges due to staff turnover and trauma
Trauma‐informed and holistic approaches to care	Understanding behaviour through trauma lens Avoiding re‐traumatisation in healthcare Person‐centred and harm‐reduction models
Need for stability in housing, healthcare, and substance use support	Housing as foundation for recovery Challenges of service continuity with instability Rejection of abstinence‐only models
Systemic and policy failures	Fragmentation across systems Crisis‐driven rather than preventative models Discharge and Mental Health Act policy issues

### 3.1 Barriers to Accessing Healthcare and Mental Health Services

A key issue identified across interviews is the significant difficulty people experiencing homelessness face in accessing healthcare and mental health services. Systemic problems, such as restrictive eligibility, poor coordination, long waits, and the instability of homelessness, create major barriers. Professionals voiced frustration at a fragmented, bureaucratic system that frequently fails to meet the needs of the most vulnerable.

A major barrier identified is the inflexibility of the healthcare system, particularly in mental health services, where rigid criteria exclude individuals who do not fit into predefined categories. Several interviewees highlighted the issue of catchment areas, which prevent homeless individuals from accessing mental health services unless they have a registered address in the area

Psychiatric services often refuse to admit homeless individuals, citing various reasons, such as catchment area restrictions. (Service Leader)

The mental health system's strict reliance on catchment areas makes it incredibly difficult to access care. Homeless people don't fit into these boundaries, and as a result, many don't receive any support. (Hospital consultant)

Another significant barrier is the exclusion of individuals with dual diagnoses (both a mental health condition and substance use disorder) from many mainstream mental health services. This exclusion leads to a cycle where individuals with substance use disorders are denied psychiatric care because of their addiction, and their mental health continues to deteriorate.

Many clients with clear signs of mental illness struggle to obtain a diagnosis because they also have an addiction, leaving them in a continuous cycle of exclusion from mental health care. (Nursing Manager)

Unless somebody is at the point of needing to be sectioned, they're not engaged with ongoing mental health support. That's a huge gap. (Service Leader)

Waiting times for psychiatric care were also identified as a major issue. Many individuals who are experiencing acute distress are often turned away because they do not meet the threshold for immediate intervention. Instead of early support, individuals are forced to wait until their condition deteriorates to a crisis point, at which stage law enforcement often becomes involved.

I often section people not because they are an immediate risk to themselves or others, but because their inability to access care means their health will deteriorate. The new Mental Health Act changes will restrict this further. (General Practitioner)

Healthcare settings themselves also pose significant barriers, as many individuals experiencing homelessness report negative past experiences with professionals, which deter them from seeking care in the future. Stigma and discrimination from healthcare workers can reinforce existing distrust in the system.

Many of my clients report feeling judged or dismissed when seeking medical care, especially if they have a history of addiction. (Psychotherapist)

We did a piece of research around people living with HIV, and their really appalling experiences at diagnosis, the way they were treated, dismissed, and belittled. It's no wonder people don't engage with healthcare services. (Service Leader)

Finally, practical barriers such as the lack of a fixed address, phone number, or necessary documentation make it difficult for individuals experiencing homelessness to access follow‐up care, medication, and referrals.

Patients are sent letters asking them to call to confirm appointments, or else the appointment is cancelled. This doesn't work for homeless people who may not have a phone, fixed address, or the literacy skills to understand the letter. (Specialist Nurse)

### 3.2 The Importance of Building Trust and Therapeutic Relationships

A recurring theme across the interviews was the fundamental role of trust in delivering healthcare and mental health support to people experiencing homelessness. Professionals noted that previous negative experiences with healthcare, social services, and law enforcement often result in deep mistrust. Without a strong therapeutic relationship, many individuals are reluctant or unable to engage with services, making trust‐building essential to effective care.

One of the key challenges in building trust is that many individuals experiencing homelessness have faced systemic failures, abandonment, and stigma. They often come into contact with services only when in crisis, and their past encounters with healthcare professionals have frequently been negative.

Many homeless people have trust issues due to a lifetime of being let down by family and professionals, so trust has to be earned. (Specialist Nurse)

Another major barrier to trust‐building is the high turnover of staff in homeless services, which makes it difficus lt for individuals to establish stable, long‐term relationships with professionals.

Every time somebody comes into one of our services, they see a whole new set of faces, and I think that can be very unsettling. It means you have to start building that relationship all over again. (Service Leader)

A major challenge is that homelessness services often experience high staff turnover, leading to inconsistent support. Clients have frequently developed relationships with key workers or other professionals, only to have those individuals leave. This can reinforce feelings of abandonment and distrust. (Psychotherapist)

Flexibility in service provision was identified as a key enabler of trust‐building. Many professionals emphasised the need to meet people where they are, rather than expecting individuals to engage with rigid or highly structured services.

If a service is structured, time‐restricted, and feels impersonal, like in a traditional health centre, then forming a strong therapeutic relationship is more challenging. (Service Leader)

Consistency and follow‐through were also highlighted as critical aspects of trust‐building. Many individuals experiencing homelessness have been repeatedly let down by professionals who promise support but fail to deliver.

Within hostels, relationship‐building is easier because I see people regularly. They might come in with respiratory issues or looking for sleep medication, and while I may not prescribe what they're looking for, I use those interactions as opportunities to talk about their general health and mental health. (General Practitioner)

Humour and informal engagement were also cited as useful strategies for breaking down initial barriers and helping individuals feel more at ease.

Humour helps as well. If someone is demanding sleeping tablets, I might joke, ‘Sure, will I throw in a few diazepam, some Lyrica, and whatever else while I'm at it?’ and they'll laugh, realising that's not going to work with me. But from there, I can steer the conversation toward what support they actually need. (General Practitioner)

Despite these strategies, some professionals noted that trust cannot always be established, particularly when individuals are dealing with severe trauma, mental illness, or are in an active crisis state.

There have been times where I simply could not build a relationship, no matter how much effort I put in. Some people are too unwell, too traumatised, or too distrustful to engage with services. In those cases, all we can do is be consistent and ensure they know the door is open when they are ready. (Psychotherapist)

### 3.3 Trauma‐Informed Care and Holistic Approaches to Mental Health

A consistent theme across the interviews was the need for trauma‐informed care when working with individuals experiencing homelessness. Many of the professionals interviewed emphasised that homelessness is rarely an isolated experience; rather, it is deeply intertwined with past trauma, mental health struggles, substance use, and systemic failures. A trauma‐informed approach acknowledges these complexities and ensures that services are designed to be flexible, non‐judgemental, and responsive to the needs of individuals with histories of trauma.

Many professionals noted that a traditional, medicalised approach to mental health care often fails to meet the needs of this population. Instead, care needs to be person‐centred and adapted to individuals who may struggle with engaging in structured services due to their past experiences of trauma and instability

If we can't acknowledge somebody's pre‐existing trauma and their life experiences before and when they come to us, I think we're at nothing. (Service Leader)

One of the key principles of trauma‐informed care is understanding behaviour as a response to trauma, rather than simply seeing it as ‘difficult’ or ‘non‐compliant’. Many interviewees pointed out that traditional healthcare models tend to focus on diagnosis and treatment plans without considering the broader context of an individual's life. This approach often alienates individuals experiencing homelessness, many of whom have had previous negative encounters with healthcare professionals, social services, and law enforcement.

Understanding the deep connection between trauma, physical illness, and mental health would have completely changed my approach to nursing. Even a half‐day training session on trauma‐informed care would be eye‐opening for many professionals. (Specialist Nurse)

Another key component of trauma‐informed care is recognising the importance of autonomy and choice in healthcare interactions. Many individuals experiencing homelessness have lived through experiences where they have had little to no control over their circumstances, and forcing them to engage with services in a rigid, structured way often leads to disengagement.

Assuming we can walk into someone's life and offer help without consent is a mistake. Respecting autonomy is critical. (Service Leader)

When someone has been traumatised repeatedly, by their family, by the system, by healthcare professionals, telling them ‘you have to do this’ is the fastest way to make them walk away. (Hospital consultant)

Many interviewees criticised the way mainstream mental health services expect individuals to be abstinent from substances before receiving care, arguing that this creates a significant barrier for those who use substances as a way to manage trauma.

The vast majority of our clients have a dual diagnosis, whether formally diagnosed or not. Many have severe mental health conditions and substance use disorders, but the system still operates as though these need to be treated separately. (Service Leader)

If we're not going to acknowledge that people self‐medicate as a way of managing trauma, then we're not really treating them. We're just setting them up to fail. (General Practitioner)

Several interviewees noted that individuals experiencing homelessness often relate more to peer support workers than to highly trained professionals because they see them as people who truly understand their experiences.

Often, individuals who have struggled in traditional mental health services benefit more from support workers with lived experience rather than highly trained professionals. (Service Leader)

Basic needs, such as housing, food, and safety, were noted as a priority, before attempting to engage individuals in mental health treatment. Without addressing these fundamental concerns, it is nearly impossible for individuals to fully engage in therapy, medication adherence, or long‐term recovery plans.

Mental well‐being is vital, but it's nearly impossible to achieve while someone is homeless. You can't talk about coping strategies when someone doesn't even know where they'll be sleeping tonight. (Service Leader)

### 3.4 The Need for Stability in Housing, Healthcare, and Substance Use Support

A recurring theme across the interviews was the fundamental role of stability in housing, healthcare, and substance use as a prerequisite for meaningful engagement with mental health services. Without stable accommodation, consistent healthcare access, and support in managing substance use, individuals experiencing homelessness face significant barriers to improving their mental well‐being. Without a foundation of stability, mental health interventions often fail to have lasting impacts, as individuals remain trapped in cycles of crisis and instability.

One of the most pressing issues raised was the disconnect between housing policies and mental health services. Many interviewees criticised the assumption that simply providing housing would be enough to support people in recovering from homelessness. Instead, they argued that housing must be accompanied by ongoing, structured support, particularly for individuals with complex mental health and addiction needs.

Housing alone isn't enough, it needs to be supported housing. Some people thrive in a structured environment, and taking them out of that and putting them into isolated settings can strip them of their social support system. (General Practitioner)

For many individuals experiencing homelessness, the lack of stability in housing leads to a breakdown in mental health and medical care. Many interviewees highlighted the challenges of maintaining treatment plans, taking medication consistently, and attending appointments when housing is unpredictable.

We can prescribe medication, but if someone has no fridge to store insulin, no regular access to food, and no safe place to sleep, then the prescription is pointless. (Specialist Nurse)

Even when someone is engaged in services, if they get moved from one hostel to another or end up rough sleeping again, they often disengage completely. Stability is everything. (Nursing Manager)

Another challenge identified was the instability in substance use patterns and the unrealistic expectations placed on individuals to achieve abstinence before accessing mental health care. Many professionals expressed frustration that services often refuse to support individuals unless they commit to complete sobriety, even though substance use is often a coping mechanism for trauma and mental illness.

We, as a society, are very fixated on abstinence, people have to stop taking drugs, and unless they stop, they can't achieve anything. But we've actually seen clients who reduce their substance use to a less chaotic amount, and that in itself is progress. (Service Leader)

It's not realistic to expect someone to stop using when their mental health is completely unstable. (Service Leader)

The rigid structures of many treatment programmes were also identified as a barrier to stability. Many individuals experiencing homelessness struggle to comply with strict rules and schedules due to the unpredictability of their daily lives, leading to high dropout rates from structured addiction and mental health services.

Many addiction services have rules that simply don't work for homeless individuals. If someone misses three appointments, they're discharged from the programme. But these are people who don't always know where they'll be sleeping that night, how can we expect them to show up every time? (General Practitioner)

Many professionals argued for a shift toward a harm‐reduction approach rather than an abstinence‐focused model, as well as greater investment in long‐term, integrated support services. Instead of expecting individuals to become completely stable before receiving care, services should focus on meeting people where they are, supporting gradual reductions in harm, and providing care that adapts to the realities of homelessness and addiction.

We need to move away from an all‐or‐nothing approach. Even small steps, like reducing alcohol use, stabilising on methadone, or attending therapy once a month … can be life‐changing for people. (Service Leader)

The most important thing is consistency. People need to know that even if they relapse, even if they miss appointments, the support will still be there when they're ready. (Specialist Nurse)

### 3.5 Systemic and Policy Failures in Addressing Homelessness and Mental Health

Many of the professionals highlighted that current systems are not designed to accommodate the complex, intersecting needs of people who are homeless, particularly those with dual diagnoses (mental illness and substance use disorder), unstable housing, or ongoing trauma histories.

A key issue raised was the fragmentation of services, where housing, healthcare, mental health, and addiction services operate in silos rather than providing integrated care. Many interviewees noted that the current system places unrealistic expectations on service users to navigate multiple bureaucratic processes, which can be overwhelming and unachievable for people experiencing homelessness.

We have a very disjointed healthcare system. Clients go all around the city to get a wound dressed here, meds there, and methadone over there. It would be impossible for somebody who's housed, working, and fully educated to keep up with that level of access to care. (Service Leader)

A major policy failure identified by multiple professionals was the exclusion of people with dual diagnoses from mental health services. Many mental health services refuse to treat individuals who are actively using substances, while addiction services often require mental health stabilisation before providing support. This leads to a cycle of exclusion where individuals are denied care from both systems.

Many clients with clear signs of mental illness struggle to obtain a diagnosis because they also have an addiction, leaving them in a continuous cycle of exclusion from mental health care. (Nursing Manager)

Another significant challenge is the reliance on crisis‐driven interventions rather than preventative care. Many professionals expressed frustration that individuals often cannot access mental health services until they reach a crisis point, at which stage police or emergency services become involved.

The system isn't built for prevention, it's built for crisis. People can be floridly psychotic, but unless they are an immediate risk to themselves or others, they won't get help. Instead, they are left to deteriorate until the police are called. (General Practitioner)

Emergency mental health services often turn people away, claiming they are ‘not unwell enough’ for intervention. This sends a devastating message to individuals who have finally built up the courage to seek help. (Psychotherapist)

Many interviewees also highlighted the failures in the discharge process from hospitals and psychiatric units, where individuals experiencing homelessness are often sent back onto the streets with no follow‐up care.

If someone is homeless and being discharged from hospital, there's no guarantee that we'll even be informed. There is no systematic follow‐up, and many people just disappear back into the streets. (Specialist Nurse)

Additionally, proposed changes to the Mental Health Act were widely seen as a policy failure that would further restrict access to psychiatric care. Many professionals warned that the new legislation would make it even harder to admit individuals for treatment, even when it was clear they needed urgent care.

The revision of the Mental Health Act is a huge concern. If I see someone who is floridly psychotic but not an immediate risk, I won't be able to intervene. Instead, they will be left to deteriorate until they reach the point where the police are called. (General Practitioner)

The criminalisation of homelessness was also identified as a growing concern, particularly as law enforcement is often used to manage individuals experiencing mental health crises instead of healthcare professionals. Many interviewees stressed that homelessness should be treated as a public health issue, not a criminal justice issue.

Right now, people see prison as a place of respite because at least they have structure, food, and shelter. That tells you everything you need to know about how badly the system is failing them. (Psychotherapist)

Several professionals criticised the lack of long‐term planning in policy responses to homelessness and mental health, noting that the focus tends to be on short‐term solutions rather than addressing the root causes of the issue.

We need a coordinated system to identify and respond to mental health crises among people experiencing homelessness. Currently, there is no clear pathway, meaning access to care is inconsistent and often dependent on location. (Service Leader)

There needs to be more community‐based crisis mental health supports and forensic community assessments. Different services often do not communicate effectively, which results in clients falling through the cracks. (Nursing Manager)

## 4 Discussion

This study critically explores how frontline healthcare professionals in Ireland manage acute mental health deterioration among people experiencing homelessness. Situated within a broader national and European crisis in housing and health inequality, the findings highlight both relational strengths and structural failures in current care approaches. Participants described a system defined by exclusion, fragmentation, and chronic under‐resourcing, where trust‐based, person‐centred care becomes not only best practice but a compensatory response to institutional neglect.

The findings reinforce established evidence on the disproportionate mental health burden among people experiencing homelessness, who are more likely to experience severe psychological distress, trauma, dual diagnoses, and untreated psychiatric conditions (Fazel et al. 2014; Gutwinski et al. 2021; Ingram et al. 2023). Systemic barriers such as catchment‐based eligibility, abstinence‐based thresholds, and rigid referral processes further limit access to appropriate care. These exclusionary mechanisms reflect international concerns about the structural misalignment of traditional mental health systems with the needs of marginalised populations (Metzl and Hansen 2014).

As a result, generalist and primary care services often bear the brunt of managing complex mental health needs, including acute crises. Participants described working beyond their remit, delivering crisis care in settings neither designed nor resourced for such interventions. This underscores how individual clinicians are left to absorb institutional shortfalls (Kulkarni et al. 2013; Wirth et al. 2019). While participants did not consistently articulate emotional distress or burnout, their accounts point to ongoing ethical ambiguity and sustained professional strain, which may place clinicians at risk of moral distress within structurally constrained care environments (Epstein and Hamric 2009).

Despite pressures, clinicians consistently described building trust and therapeutic relationships as a core strength; seen as critical to engaging individuals with complex trauma histories, many of whom were sceptical of institutional care. Practices such as consistency, humour, flexibility, and non‐judgement were central to this work and closely aligned with trauma‐informed care principles emphasising safety, choice, respect, and collaboration (Center for Substance Abuse Treatment (US) 2014). However, high staff turnover, fragmented services, and unstable housing arrangements often undermined this trust.

Participants consistently endorsed trauma‐informed, holistic, and harm‐reduction approaches, rejecting narrow, medicalised models in favour of integrated care that addresses physical health, substance use, social connection, and housing. There was widespread frustration with abstinence‐based thresholds that exclude those actively using substances, particularly as substance use was often viewed as an adaptive response to trauma (Padgett et al. 2008). Trauma was seen as central to many clinical presentations, often originating early in life and compounded by homelessness and service exclusion (Muskett 2014). Yet, current healthcare systems were described as ill‐equipped to respond effectively, with rigid assessments and coercive practices risking re‐traumatisation and further disengagement (Emsley et al. 2022; Warren et al. 2021).

Participants also emphasised the foundational role of stability in housing, healthcare, and substance use support, noting that instability in any area could disrupt care. Transitions in housing frequently led to disengagement, underscoring the need for continuity. While international models like Housing First show promise (O'Campo et al. 2022), the lack of wraparound services in Ireland was seen as a major gap. Housing alone was considered insufficient without structured, ongoing support for individuals with complex needs.

Fragmentation across housing, healthcare, mental health, and addiction services was a recurring concern. Siloed systems placed unrealistic burdens on individuals to coordinate their own care. The absence of joined‐up discharge planning, the over‐reliance on emergency departments and policing for crisis response, and the growing criminalisation of homelessness all signal a systemic failure to meet vulnerable people's needs. These concerns echo critiques of Ireland's health system as overly reactive and insufficiently preventative (Siersbaek et al. 2023) and underline the need for integrated, person‐centred care models.

Consistent with growing international evidence, the findings of this study also point to the value of co‐designed and participatory approaches in addressing the misalignment between health services and the needs of people experiencing homelessness. Participants described care environments and pathways that were often inflexible, fragmented, and poorly attuned to the realities of homelessness, reinforcing concerns raised by Carmichael et al. (2023) regarding the structural mismatch between mainstream health systems and the lived experiences of marginalised populations. Co‐design offers a mechanism through which people with lived experience of homelessness, alongside frontline professionals, can actively shape services that are accessible, responsive, and grounded in real‐world need. Evidence from a recent systematic review by Schiffler et al. (2023) demonstrates that co‐designed mental health interventions for people experiencing homelessness are associated with improvements in mental health outcomes, quality of life, and reduced reliance on emergency care. These findings suggest that the meaningful involvement of people experiencing homelessness in service design is not merely aspirational but central to the development of effective, sustainable, and equitable models of mental health care.

Proposed reforms to the Mental Health Act were viewed with concern. Participants feared that changes to involuntary admission criteria would narrow access to care for individuals in crisis, particularly those who fall outside traditional risk definitions. While the reforms aim to protect rights, the lack of viable community alternatives means more individuals may be left without support.

Ultimately, this study amplifies the voices of frontline clinicians navigating Ireland's dual mental health and housing crises. Their accounts reveal a strong commitment to values‐driven, patient‐centred care, but also deep frustration with a system that repeatedly undermines their efforts. These findings align with and strengthen calls for reform grounded in equity, trauma‐informed practice, and social justice (McNeill et al. 2022; Ingram et al. 2023).

## 5 Limitations

This study is based on interviews with healthcare professionals and does not include the perspectives of people with lived experience of homelessness. As a qualitative study, findings are not generalisable but offer in‐depth insights into professional experiences within specific service contexts. Additionally, the sample may reflect individuals more engaged or experienced in homelessness and mental health care.

## 6 Conclusion

This study highlights the critical role that frontline healthcare professionals play in supporting people experiencing homelessness with acute or crisis mental health needs, often within a system that is fragmented, under‐resourced, and not designed for complexity. Participants described working beyond their formal roles to bridge systemic gaps through relational, trauma‐informed, and person‐centred care. However, the structural barriers outlined in this study continue to undermine equitable access to mental health support. These challenges are compounded by widespread stigma and discrimination, which foster mistrust and disengagement from care.

The findings underscore the urgent need for trauma‐informed, integrated approaches that prioritise stability in housing, substance use, and healthcare access. Embedding services in trusted, low‐threshold settings, investing in staff training, and amplifying the voices of those with lived experience are essential to creating responsive and humane models of care. Nurses, in particular, are well‐positioned to lead such innovations.

While grounded in the Irish healthcare context, the challenges described in this study reflect structural conditions evident across many high‐income countries, where homelessness, service fragmentation, and mental health inequities intersect. As levels of homelessness continue to rise internationally, the moral imperative for system‐wide reform extends beyond national borders. Ensuring that people experiencing homelessness can access timely, respectful, and effective mental health care is not only a clinical priority, but a matter of social justice.

## Ethics Statement

Ethical approval was received for this study from University College Dublin Human Research Ethics Committee REF LS‐LR‐23‐25‐Gilmore.

## Conflicts of Interest

The authors declare no conflicts of interest.

## Supporting information


**Data S1:** jpm70116‐sup‐0001‐DataS1.docx.

## Data Availability

The data that support the findings of this study are not publicly available due to the sensitive nature of the material, which includes potentially identifiable information relating to participants and specific organisations and services.
