# Supplementary material for: Clinicians' Experiences in Responding to Mental Health Crisis Among People Experiencing Homelessness in Ireland
Source: J Psychiatr Ment Health Nurs. 2026 Mar 3;33(3):434–44. doi: 10.1111/jpm.70116 (PMC13139990; doi:10.1111/jpm.70116)
Supplement: Supplementary file 1 — Data S1: jpm70116‐sup‐0001‐DataS1.docx. [file JPM-33-434-s001.docx]

Supplementary material 1: Interview schedule

**REM-CSI Interview Schedule**

1. Can you please tell me a little bit about yourself, your current role, the type of organisation you work with and what your role is in terms of health or social care for those experiencing homelessness.
2. One of the things research tells us is about the importance of relationship building with clients, can you tell me a bit about your experience of this? Are there any specific things that make this easier or harder to do?
3. Do you have any examples of where you were able or were unable to establish this kind of relationship?
4. Can you tell me a little bit about the kinds of training and education that you think would be helpful to support the care of people who are homeless experiencing acute mental health distress?
5. What have you found to be the wider determinants of mental well-being, crisis management and general health for people experiencing homelessness?
6. Could you prioritise them, which is the most important in your opinion?
7. Are there any things that you find helpful in addressing these determinants in your practice? Are there any things that you see as barriers to addressing these determinants
8. Are specificities about the organisation that you work with which make it easier, or more difficult for you to provide appropriate care to those experiencing homelessness who are in a mental health crisis?
9. Can you talk to me about any regulatory or policy barriers or enablers you see to provide appropriate care to those experiencing homelessness who are in a mental health crisis?
10. Is there anything else you’d like to add or think we haven’t addressed in terms of providing crisis mental health support for those experiencing homelessness?
